# Body mass index and physical fitness in university students: sex differences and nonlinear patterns in a large cross-sectional study

**DOI:** 10.7717/peerj.21656

**Published:** 2026-08-11

**Authors:** Qiang Wei, Lei Wang, Chun Yang, Wei Sun, Siqin Shen

**Affiliations:** 1 Department of Physical Education, Tangshan Normal University, Tangshan, Hebei, China; 2 College of Physical Education, Dalian University, Dalian, Liaoning, China

**Keywords:** Body mass index, Physical fitness, Nonlinear associations, Sex differences, University students, Cross-sectional study

## Abstract

**Background:**

Body mass index (BMI) is routinely used in university health surveillance, but its sex- and grade-specific association with multidimensional physical fitness, including nonlinear patterns, remains unclear.

**Methods:**

Data from 16,292 university students (8,505 females; 7,787 males; aged 17–23 years) who completed the national standardized fitness assessment were analyzed in this cross-sectional study. Vital capacity, 50-m sprint, standing long jump, sit-and-reach, 800/1,000-m run, and muscular endurance were standardized within sex to derive a Physical Fitness Index (PFI), with time-based tests reverse-scored. Two-way analyses of variance examined associations of sex, academic grade, and BMI category with PFI. Sex- and grade-specific quadratic models were used to characterize BMI–PFI curves and estimate the model-derived BMI value associated with peak predicted PFI. Pearson correlations assessed associations between PFI and individual fitness components.

**Results:**

Academic grade was associated with PFI, whereas no overall sex effect was observed (sex: *F* (1, 16,284) = 1.51, *p* = 0.219, partial η^2^ = 0.000093; grade: *F* (3, 16,284) = 74.54, *p* < 0.001, partial η^2^ = 0.0136). The sex × grade interaction was statistically significant but small (*F* (3, 16,284) = 20.44, *p* < 0.001, partial η^2^ = 0.00375). Within both sexes, normal-weight students showed the highest PFI; underweight students had higher PFI than overweight and obese peers, while PFI was lower in the overweight and obese groups. The BMI–PFI association was stronger in males (partial η^2^ = 0.1061) than in females (partial η^2^ = 0.0287). All quadratic models showed a concave BMI–PFI pattern, with lower predicted PFI at higher BMI values and model-derived peak estimates varying across sex–grade subgroups. These estimates should not be interpreted as physiological optima or recommended BMI targets. Correlations indicated that PFI integrated cardiorespiratory fitness, lower-limb power, flexibility, muscular endurance, and running performance.

**Conclusion:**

BMI was associated with multidimensional physical fitness in this large cohort of university students, with lower PFI observed at both low and high BMI values. BMI showed a stronger association with PFI than academic grade, particularly in males. These findings support BMI-informed and sex-sensitive fitness monitoring, but should be interpreted as cross-sectional associations because body-composition and behavioral measures were unavailable.

## Introduction

Physical fitness is an important marker of current and future health in young people, reflecting multiple physiological capacities rather than a single performance attribute. Large-scale surveillance work has documented secular declines in cardiorespiratory fitness and muscular performance among youth over recent decades, raising concern about population-level fitness trajectories (Tomkinson et al., 2017). In parallel, evidence from longitudinal and cross-sectional studies indicates that higher fitness in adolescence and young adulthood is associated with more favorable adiposity, cardiometabolic profiles, and lower morbidity later in life (Carnethon et al., 2003; Ortega et al., 2008). University students represent a population of particular interest, as the transition from secondary school to higher education is often accompanied by reduced structured physical activity, increased sedentary time, and lifestyle changes that may be related to less favorable weight status and fitness (Haase et al., 2004; Guthold et al., 2018). Because fitness includes cardiorespiratory endurance, muscular strength and endurance, flexibility, speed, and power, relying on a single test item may provide only a partial view of health-related physical fitness. Composite approaches that integrate multiple fitness domains have therefore been recommended for population-level assessment and surveillance (Ortega et al., 2008; Ruiz et al., 2011).

Body mass index (BMI) is widely used in health surveillance to classify weight status, yet its association with physical fitness in young people is complex. Numerous studies in children and adolescents report that overweight and obese individuals tend to perform worse in endurance running, sprinting, and jumping tasks than their normal-weight peers, while underweight youth may show reduced muscular strength and power (Lopes et al., 2017; Chen et al., 2022; Gómez-Campos et al., 2025). Several large cross-sectional studies have further suggested that the association between BMI and fitness may be nonlinear, with both low and high BMI linked to poorer performance and higher fitness often observed near the normal-weight range (Zhao et al., 2012; Kwieciński et al., 2018; Chen et al., 2022). However, most available evidence comes from school-age populations, and findings in university students remain comparatively limited. Recent work in Chinese university cohorts has confirmed associations between higher BMI and poorer performance in several fitness indicators, but these studies have largely relied on linear models or BMI-category comparisons rather than examining the continuous shape of the BMI–fitness association across the full BMI range (Wu et al., 2024; Li, Shi & Tao, 2025). As a result, it remains unclear whether the BMI–fitness relationship in university students is adequately described by categorical differences alone, or whether a curvilinear pattern can be observed when BMI is treated as a continuous variable.

A further limitation of previous research is that individual fitness components are often analyzed separately. Although this approach is useful for identifying domain-specific performance differences, it does not provide an integrated estimate of multidimensional fitness. This distinction is important because BMI may relate differently to weight-bearing tasks, cardiorespiratory endurance, flexibility, and muscular endurance. A composite Physical Fitness Index (PFI) based on standardized component scores can summarize these related but distinct domains into a single surveillance-oriented indicator while retaining the contribution of each test item. Such an index is particularly useful in large-scale university health monitoring, where the aim is not to replace individual test interpretation but to characterize overall fitness patterns across sex, grade, and weight-status groups.

Subgroup differences in the BMI–fitness relationship are also not fully understood. Sex-related contrasts in body composition, muscle mass, fat distribution, and habitual activity may lead to different BMI–fitness gradients in males and females (Hunter & Senefeld, 2024; Joyner, Hunter & Senefeld, 2025). In addition, university students’ weight status and fitness are not static across their academic progression. Longitudinal evidence suggests that the first years at university are often accompanied by increases in body mass and unfavorable changes in body composition, together with modest declines in fitness (Deliens et al., 2015). Yet grade-related patterns in BMI and fitness have been explored in only a small number of studies, typically with limited sample sizes or without explicitly testing whether BMI–fitness curves differ by sex and academic year. Therefore, sex- and grade stratified nonlinear analyses may provide a more detailed understanding of whether BMI-related fitness patterns are consistent across university subgroups or vary according to demographic and academic-stage characteristics.

The present study analyzed data from 16,292 university students who completed a standardized national fitness assessment. Building on previous categorical and linear analyses, this study aimed to advance current evidence by combining a sex-standardized multidimensional PFI with sex- and grade-specific nonlinear modeling of the BMI–PFI association. The specific aims were to (1) examine sex- and grade-related differences in multidimensional physical fitness, (2) compare PFI across BMI categories within each sex, and (3) characterize nonlinear relationships between BMI and PFI using sex- and grade-specific quadratic models to estimate model-derived BMI values associated with peak predicted PFI, rather than to define physiological optima or recommended BMI targets.

## Materials and Methods

### Ethics approval and informed consent

This study was approved by the Medical Ethics Committee of Tangshan Normal University, Tangshan, China (Protocol No. TSNU-PE-2024-001; approval date: 15 March 2024) and was conducted in accordance with the Declaration of Helsinki. This study was based on routine physical fitness testing records and was approved as existing-data research. All data used for analysis were de-identified prior to use. All participating students were informed about the aims and procedures of the study and provided written informed consent for the use of their de-identified data for research and publication.

### Participants

A total of 16,292 university students (8,505 females and 7,787 males), aged 17–23 years, were included in the analysis. All participants completed the standardized annual physical fitness assessment as part of the national student fitness testing program. The analysis was based on de-identified institutional records from students with complete anthropometric data and complete results for all six required fitness components. Records were retained only when sex, academic grade, age, height, body mass, BMI, and all fitness test results were available.

Because this study used an existing de-identified testing dataset, participant selection was based on the availability of complete valid records. No additional health-related screening information was available in the dataset. Specifically, the dataset did not record whether students had medical conditions, recent musculoskeletal injuries, habitual physical activity levels, sedentary behavior, dietary intake, sleep quality, or training status. Therefore, participants could not be excluded on the basis of these factors, and these variables could not be included as covariates in the statistical models. No additional inclusion or exclusion criteria were applied beyond the completeness of the dataset and availability of the variables required for the present analyses.

### Anthropometric measures

Body height and mass were measured using calibrated stadiometers and digital scales. BMI was calculated as weight divided by height squared (kg/m^2^). According to the Chinese adult BMI classification criteria, participants were classified into four BMI categories: underweight (BMI < 18.5 kg/m^2^), normal weight (18.5 ≤ BMI < 24.0 kg/m^2^), overweight (24.0 ≤ BMI < 28.0 kg/m^2^), and obese (BMI ≥ 28.0 kg/m^2^). All measurements were obtained in a standardized laboratory environment by certified examiners to minimize inter-rater variability.

### Physical fitness assessment

Six components of physical fitness were evaluated according to the national standardized protocol: vital capacity (VC), 50-m sprint, standing long jump (LJ), sit-and-reach flexibility, a gender-specific middle-distance run (800 m for females and 1,000 m for males), and muscular endurance (sit-ups for females and pull-ups for males). All tests were administered by certified examiners using calibrated equipment within the same seasonal period to minimize environmental variability.

### Physical fitness index

To construct the PFI, each fitness component was standardized within sex using z-scores:

$${Z_i} = \displaystyle{{{x_i} - \bar x} \over {SD}},$$

because shorter times indicate better performance, sprint and endurance-run times were multiplied by −1 prior to standardization. The PFI was calculated as the sum of the six standardized component scores:

$PFI = {Z_{VC}} + {Z_{50\,m}} + {Z_{LJ}} + {Z_{SitReach}} + {Z_{RunTime}} + {Z_{StrengthReps}}.$

This unit-weighted method follows national testing practice and avoids model-dependent weighting biases. Higher PFI values indicate better overall physical fitness.

This z-score summation approach has been used in previous large-scale studies examining the association between BMI and Physical Fitness Index in Chinese children, adolescents, and university students (Bi et al., 2019; Sun et al., 2022; Li et al., 2023). In these studies, multiple field-based fitness components were standardized and combined to provide an integrated indicator of overall physical fitness, with time-based tests reverse-scored before summation. In the present study, the PFI was therefore used as a surveillance-oriented composite index to summarize multidimensional fitness across cardiorespiratory function, speed, lower-limb power, flexibility, endurance running performance, and muscular endurance.

Sex-specific standardization was applied because males and females completed partly different muscular endurance and middle-distance running tests and because raw performance levels differ substantially by sex. This approach allowed the PFI to reflect relative multidimensional fitness within sex rather than absolute between-sex performance differences. Equal weighting was retained because all six components are included in the standardized national fitness assessment and represent complementary domains of health-related physical fitness. The PFI was not intended to replace interpretation of individual fitness components; rather, it was used to provide an overall index for examining BMI-related and grade-related patterns in a large university cohort.

### Statistical analysis

Descriptive statistics are presented as mean ± standard deviation (SD) for BMI and PFI across sex, grade, and BMI categories. Group differences in PFI were examined using two-way analysis of variance (ANOVA) with sex and grade as factors. Separate two-way ANOVAs were then conducted within each sex, with BMI category and academic grade as factors.

Assumptions of normality and homogeneity of variance were evaluated using the Shapiro–Wilk and Levene tests, respectively. Normality was examined within the analysis cells, and the Shapiro–Wilk results were interpreted together with distributional summaries because large samples may detect minor deviations from normality. Homogeneity of variance was evaluated before ANOVA-based group comparisons, and the results of assumption testing are reported in the Results section. Effect sizes were reported as partial eta squared (partial η^2^). Given the large sample size, statistical significance was interpreted together with effect-size magnitude to distinguish statistically detectable differences from differences with limited practical relevance.

Pearson correlation coefficients (two-tailed) were computed to examine associations among PFI and the six fitness components. Correlations were visualized using a color-coded matrix.

To further examine whether the fitness components showed problematic multicollinearity or an interpretable underlying structure, additional exploratory analyses were conducted using the six sex-standardized and performance-oriented component scores used to construct the PFI. Pairwise correlations and variance inflation factors (VIFs) were examined to assess multicollinearity among components. An exploratory principal component analysis (PCA) was then performed on the component correlation matrix. Sampling adequacy was assessed using the Kaiser–Meyer–Olkin (KMO) statistic, and Bartlett’s test of sphericity was used to determine whether the correlation matrix was suitable for structure analysis. This analysis was used to describe the internal structure of the component set rather than to redefine the PFI weighting scheme.

### Nonlinear modeling of the BMI–PFI association

To characterize potential nonlinear associations between BMI and PFI, second-order polynomial regression models were fitted separately for each sex and grade:

$PFI = {\beta _0} + {\beta _1}\left( {BMI} \right) + {\beta _2}\left( {BM{I^2}} \right) + \varepsilon,$

where PFI = Physical Fitness Index, ${\beta _0}$ = intercept, ${\beta _1}$ = linear BMI coefficient, ${\beta _2}$ = quadratic BMI coefficient, and ε represents the error term.

Quadratic regression was selected *a priori* because previous BMI–fitness studies have reported curvilinear or inverted-U-shaped associations, and because this model provides a parsimonious and interpretable approach for testing whether PFI increases up to a peak and then declines at higher BMI values. The purpose of this analysis was explanatory rather than predictive. Therefore, the quadratic model was used to summarize the overall shape of the BMI–PFI association across predefined sex–grade subgroups, rather than to establish clinical BMI thresholds or physiological target values. More flexible approaches, such as restricted cubic splines or generalized additive models, may capture local nonlinear variations, but they were not selected as the primary analysis because the present study focused on an interpretable single-peak pattern.

A significant negative quadratic coefficient ( ${\beta _2} < 0$) was interpreted as evidence of a concave BMI–PFI pattern, indicating that predicted PFI increased up to a model-derived peak and then declined at higher BMI values. Separate models were fitted for each sex and academic grade (four grades × two sexes), resulting in eight independent regression models.

### Estimation of BMI associated with peak predicted PFI

For each sex–grade subgroup, the BMI value at which predicted PFI reached its maximum was analytically derived as:

$$BM{I_{peak}} = - \displaystyle{{{\beta _1}} \over {2{\beta _2}}},$$

where $BM{I_{peak}}$ represents the model-derived BMI value associated with peak predicted PFI in the fitted quadratic curve. This value was interpreted as a statistical vertex estimate rather than a physiological optimum or recommended BMI target.

Predicted PFI values were generated at 100 equally spaced BMI points within the observed BMI range for each subgroup, and these values were used to construct BMI–PFI curves. Statistical significance was set at *p* = 0.05. Reporting of this cross-sectional study followed the Strengthening the Reporting of Observational Studies in Epidemiology (STROBE) guideline (File S1).

### Data availability

The de-identified dataset analyzed during the current study is provided as File S2.

## Results

### Participant characteristics

Across the full sample, female students in Grades 1–4 ranged in age from 17.5 to 20.6 years, and male students from 17.6 to 20.8 years. Mean BMI values fell within the normal range for both sexes and showed only minor variation across grades. Descriptively, males had higher values for vital capacity, standing long jump, muscular endurance, and better sprint and endurance running performance, whereas sex differences in flexibility were smaller.

Within each sex, descriptive values for individual fitness components varied across grades but did not show a consistent directional pattern. For example, endurance-run time increased slightly with grade in both sexes, indicating slower performance, while strength repetitions, sprint performance, and flexibility exhibited fluctuations without a clear trend. Because individual components were not analyzed inferentially, these variations should be interpreted descriptively rather than as evidence of systematic grade-related change.

Overall, the descriptive profiles reflected well-established sex differences in physical fitness and modest grade-related variation within each sex group (Table 1).

**Table 1:** Descriptive characteristics of participants by sex and academic grade. Values are presented as mean ± standard deviation.

Sex	Grade	*N*	Age (yr)	BMI (kg/m^2^)	VC (mL)	Sprint50 (s)	LJ (cm)	SitReach (cm)	RunTime (s)	Strength (reps)	PFI
Female	1	2,375	17.49 ± 0.76	21.78 ± 3.50	2,740.12 ± 690.16	9.85 ± 1.05	149.36 ± 17.96	14.48 ± 6.71	259.37 ± 29.64	29.71 ± 8.56	0.33 ± 3.27
	2	2,226	18.50 ± 0.78	21.57 ± 3.55	2,729.51 ± 675.42	10.13 ± 1.09	149.98 ± 17.57	13.66 ± 6.85	271.85 ± 35.51	28.00 ± 9.10	−0.59 ± 3.27
	3	2,017	19.56 ± 0.84	21.07 ± 3.53	2,845.34 ± 621.13	10.18 ± 1.09	154.45 ± 19.08	15.85 ± 6.88	271.46 ± 43.34	30.91 ± 8.32	0.45 ± 3.27
	4	1,887	20.62 ± 0.82	21.25 ± 3.57	2,759.00 ± 675.13	10.11 ± 1.05	149.78 ± 19.41	14.99 ± 6.34	274.41 ± 28.27	29.82 ± 7.39	−0.21 ± 3.06
Male	1	2,512	17.62 ± 0.84	22.87 ± 4.08	4,378.77 ± 889.08	7.80 ± 0.84	204.33 ± 23.53	11.51 ± 8.08	268.85 ± 37.87	4.62 ± 4.92	0.55 ± 3.42
	2	1,841	18.62 ± 0.81	23.07 ± 4.38	4,336.78 ± 946.67	7.96 ± 0.77	205.64 ± 22.69	10.21 ± 8.01	283.32 ± 41.47	4.46 ± 4.84	−0.13 ± 3.26
	3	1,787	19.68 ± 0.86	22.97 ± 4.49	4,481.31 ± 842.16	8.17 ± 0.93	209.84 ± 27.09	10.83 ± 7.68	290.98 ± 59.75	5.22 ± 4.96	0.03 ± 3.68
	4	1,647	20.75 ± 0.91	23.49 ± 4.29	4,396.76 ± 918.08	8.15 ± 0.92	205.18 ± 26.60	10.02 ± 7.12	302.49 ± 42.40	4.44 ± 4.56	−0.73 ± 3.38

**Note:**

*N*, number of participants; BMI, body mass index; VC, vital capacity; Sprint50, 50-m sprint; LJ, standing long jump; SitReach, sit-and-reach; RunTime, endurance running time; reps, repetitions; strength, sit-ups for females and pull-ups for males; PFI, physical fitness index.

### Assumption testing

Assumption testing was conducted for the ANOVA models. Shapiro–Wilk tests indicated statistically significant deviations from normality in several analysis cells. For the sex × grade model, six of eight cells showed significant Shapiro–Wilk results, with W values ranging from 0.969 to 0.999. For the female BMI category × grade model, 11 of 16 cells showed significant Shapiro–Wilk results, with W values ranging from 0.960 to 0.999. For the male BMI category × grade model, 7 of 16 cells showed significant Shapiro–Wilk results, with W values ranging from 0.973 to 0.999. These findings were interpreted cautiously because the large sample size increases sensitivity to minor deviations from normality. Distributional summaries did not indicate extreme asymmetry or kurtosis across the analysis cells, with skewness ranging from −0.90 to 0.34 and excess kurtosis ranging from −0.29 to 3.15.

Levene’s tests indicated heterogeneity of variance for the sex × grade model, *F* (7, 16,284) = 8.96, *p* < 0.001, and for the male BMI category × grade model, *F* (15, 7,771) = 1.71, *p* = 0.042. The homogeneity of variance assumption was not rejected for the female BMI category × grade model, *F* (15, 8,489) = 1.53, *p* = 0.085. Therefore, the ANOVA findings were interpreted together with partial η^2^ values, with particular caution applied to statistically significant effects with very small effect sizes.

### Sex- and grade-related differences in PFI

Table 2 presents the two-way ANOVA results for PFI by sex and academic grade. A two-way ANOVA showed a significant main effect of academic grade on PFI, *F* (3, 16,284) = 74.54, *p* < 0.001, partial η^2^ = 0.0136, but no significant main effect of sex, *F* (1, 16,284) = 1.51, *p* = 0.219, partial η^2^ = 0.000093. The sex × grade interaction was significant, *F* (3, 16,284) = 20.44, *p* < 0.001, partial η^2^ = 0.00375. In males, mean PFI values fluctuated around zero, with slightly lower values in Grades 2–4 than in Grade 1. In females, the lowest PFI was observed in Grade 2, whereas Grades 1, 3, and 4 showed relatively comparable values. Although statistically significant, the grade effect and sex × grade interaction had small partial η^2^ values, especially for the interaction term. Therefore, these findings should be interpreted as modest group-level variations rather than practically large effects.

**Table 2:** Two-way analysis of variance examining the effects of sex and academic grade on the physical fitness index. Effect sizes are reported as partial η^2^.

Factor	df	SS	*F*-value	*p*-value	Partial η^2^
Sex	1, 16,284	16.75	1.51	0.219	0.000093
Grade	3, 16,284	2,475.82	74.54	<0.001	0.0136
Sex × grade	3, 16,284	678.74	20.44	<0.001	0.00375
Error	16,284	180,278.30	–	–	–

**Note:**

df, degrees of freedom; SS, sum of squares; partial η^2^, partial eta squared.

### BMI-category differences in fitness performance

Table 3 presents PFI values across BMI categories and grades for female and male students. Across both sexes, a consistent descriptive pattern was observed, with the normal-weight group showing the highest PFI and lower scores observed in the overweight and obese groups. Among females, PFI values in the normal-weight group were consistently positive across grades, whereas overweight and obese groups showed uniformly negative values. Underweight females outperformed the overweight and obese groups across all grades, although their mean PFI fluctuated without a consistent grade-related pattern.

**Table 3:** Physical fitness index across body mass index categories and academic grades in female and male students. Values are presented as mean ± standard deviation.

Sex	Grade	BMI category	*N*	BMI (kg/m^2^)	PFI
Female	1	Underweight	364	17.55 ± 0.75	0.25 ± 3.07
		Normal	1,479	20.98 ± 1.46	0.71 ± 3.25
		Overweight	396	25.56 ± 1.10	−0.25 ± 3.02
		Obese	136	30.84 ± 2.75	−1.87 ± 3.51
	2	Underweight	367	17.46 ± 0.87	−0.53 ± 3.41
		Normal	1,425	20.93 ± 1.46	−0.35 ± 3.19
		Overweight	318	25.60 ± 1.11	−1.05 ± 3.09
		Obese	116	31.29 ± 3.47	−2.57 ± 3.59
	3	Underweight	442	17.34 ± 0.91	0.61 ± 3.21
		Normal	1,247	20.76 ± 1.48	0.75 ± 3.19
		Overweight	229	25.63 ± 1.10	−0.45 ± 3.09
		Obese	99	31.08 ± 2.92	−1.87 ± 3.62
	4	Underweight	384	17.45 ± 0.90	−0.17 ± 3.07
		Normal	1,160	20.73 ± 1.44	−0.03 ± 2.96
		Overweight	235	25.67 ± 1.10	−0.53 ± 3.04
		Obese	108	30.75 ± 2.75	−1.60 ± 3.60
Male	1	Underweight	291	17.40 ± 0.89	0.61 ± 3.13
		Normal	1,361	21.13 ± 1.53	1.28 ± 3.31
		Overweight	554	25.65 ± 1.11	0.21 ± 3.10
		Obese	306	30.77 ± 2.31	−2.14 ± 3.32
	2	Underweight	211	17.39 ± 0.90	0.35 ± 2.84
		Normal	1,010	21.21 ± 1.50	0.62 ± 3.10
		Overweight	373	25.73 ± 1.15	−0.77 ± 3.05
		Obese	247	31.51 ± 2.73	−2.68 ± 3.08
	3	Underweight	247	17.32 ± 0.90	0.77 ± 3.24
		Normal	949	21.23 ± 1.55	0.77 ± 3.56
		Overweight	345	25.72 ± 1.13	−0.28 ± 3.30
		Obese	246	31.51 ± 2.76	−3.09 ± 3.33
	4	Underweight	162	17.37 ± 0.97	−0.59 ± 3.28
		Normal	831	21.34 ± 1.54	0.06 ± 3.24
		Overweight	407	25.75 ± 1.10	−1.14 ± 3.12
		Obese	247	31.03 ± 2.56	−2.79 ± 3.37

**Note:**

*N*, number of participants; BMI, body mass index; PFI, physical fitness index. BMI categories were defined according to Chinese adult BMI classification criteria.

A similar but more marked pattern appeared in males. Normal-weight males demonstrated the strongest performance in every grade, with Grade 1 showing the greatest positive scores. Overweight and obese males exhibited substantially lower PFI scores, with the lowest values observed in Grades 3 and 4, and underweight males, although outperforming overweight and obese groups, remained below the normal-weight group.

**Figure 1:**
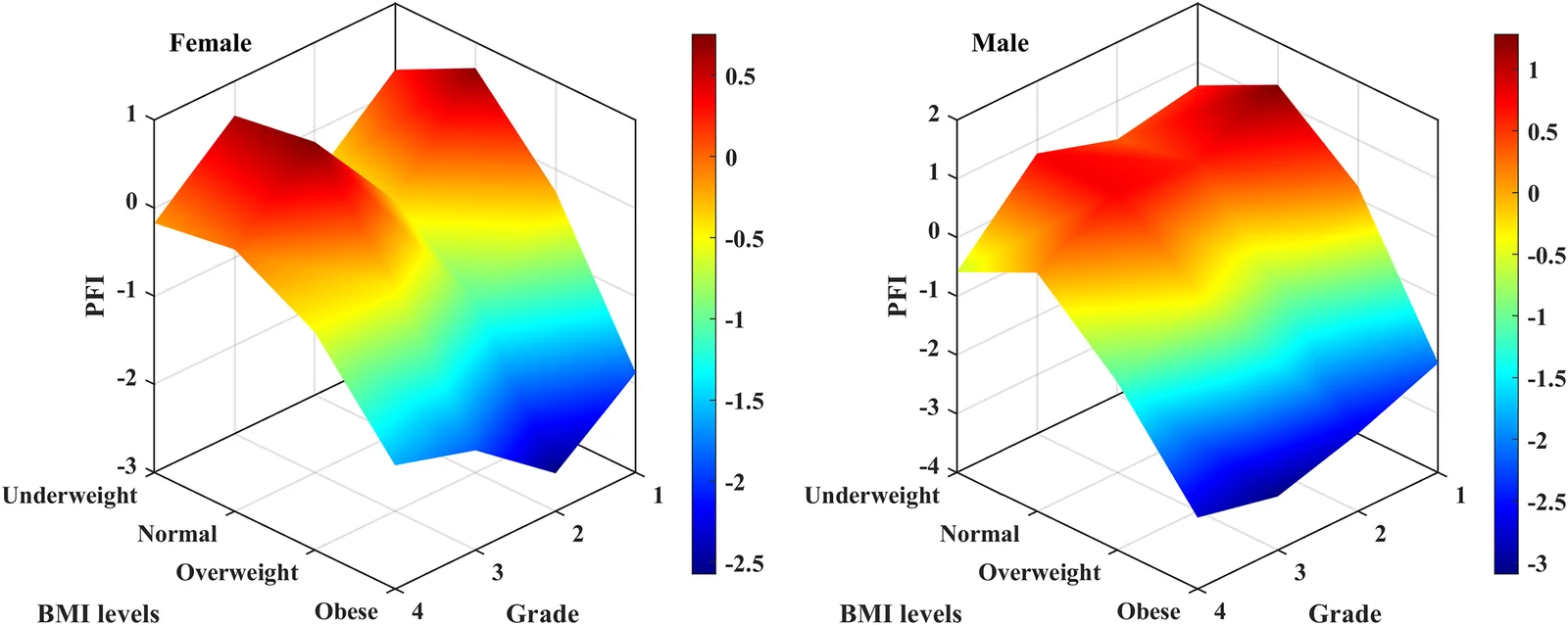
Three-dimensional response surfaces of the physical fitness index (PFI) across BMI categories and grade levels in female and male students.

Figure 1 illustrates these patterns in three-dimensional response surfaces. In both sexes, PFI declined progressively from normal weight toward overweight and obesity, and a modest downward shift across grades was also apparent. The male surface showed a more pronounced BMI-related gradient than the female surface, consistent with the larger BMI-category effect size observed in males.

Two-way ANOVAs were conducted separately for female and male students to examine the effects of BMI category and academic grade on PFI (Table 4). Among females, BMI category showed a significant main effect on PFI, *F* (3, 8,489) = 83.49, *p* < 0.001, partial η^2^ = 0.0287. Bonferroni-adjusted *post-hoc* comparisons indicated that the normal-weight group had higher PFI than the overweight and obese groups (both *p* < 0.001), and the underweight group also had higher PFI than the overweight and obese groups (both *p* < 0.001). In addition, overweight females had higher PFI than obese females (*p* < 0.001). Academic grade also showed a significant main effect, *F* (3, 8,489) = 16.98, *p* < 0.001, partial η^2^ = 0.0060, with Grade 1, Grade 3, and Grade 4 showing higher PFI than Grade 2 (all *p* < 0.001). The BMI × grade interaction was not significant, *F* (9, 8,489) = 1.51, *p* = 0.138, partial η^2^ = 0.0016, indicating that the association between BMI category and PFI was relatively consistent across academic grades in female students.

**Table 4:** Two-way analysis of variance results for physical fitness index across body mass index categories and academic grades in female and male students, with Bonferroni-adjusted *post-hoc* comparisons.

Sex	Factor	df	*F*-value	*p*-value	Partial η^2^	Significant pairwise differences (Bonferroni)
Female	BMI category	3, 8,489	83.49	<0.001	0.0287	Normal > overweight (*p* < 0.001); normal > obese (*p* < 0.001); underweight > overweight (*p* < 0.001); underweight > obese (*p* < 0.001); overweight > obese (*p* < 0.001)
	Grade	3, 8,489	16.98	<0.001	0.0060	Grade 1 > Grade 2 (*p* < 0.001); Grade 3 > Grade 2 (*p* < 0.001); Grade 4 > Grade 2 (*p* < 0.001)
	BMI × grade	9, 8,489	1.51	0.138	0.0016	Not applicable; interaction not significant
Male	BMI category	3, 7,771	307.52	<0.001	0.1061	Normal > underweight (*p* = 0.006); normal > overweight (*p* < 0.001); normal > obese (*p* < 0.001); underweight > overweight (*p* < 0.001); underweight > obese (*p* < 0.001); overweight > obese (*p* < 0.001)
	Grade	3, 7,771	27.62	<0.001	0.0106	Grade 1 > Grade 2 (*p* < 0.001); Grade 1 > Grade 3 (*p* = 0.001); Grade 1 > Grade 4 (*p* < 0.001); Grade 2 > Grade 4 (*p* = 0.001); Grade 3 > Grade 4 (*p* < 0.001)
	BMI × grade	9, 7,771	2.42	0.010	0.0028	No *post-hoc* pairwise comparisons were reported for the interaction term; the effect size was very small.

**Note:**

BMI, body mass index; df, degrees of freedom; partial η^2^, partial eta squared. BMI categories were defined according to Chinese adult BMI classification criteria.

Among males, BMI category showed a stronger main effect on PFI, *F* (3, 7,771) = 307.52, *p* < 0.001, partial η^2^ = 0.1061. Bonferroni-adjusted comparisons showed that the normal-weight group had higher PFI than the underweight (*p* = 0.006), overweight (*p* < 0.001), and obese groups (*p* < 0.001). The underweight group also had higher PFI than the overweight and obese groups (both *p* < 0.001), and the overweight group had higher PFI than the obese group (*p* < 0.001). Academic grade showed a significant main effect, *F* (3, 7,771) = 27.62, *p* < 0.001, partial η^2^ = 0.0106, with Grade 1 showing higher PFI than Grades 2, 3, and 4, and Grades 2 and 3 showing higher PFI than Grade 4. The BMI × grade interaction was also significant, *F* (9, 7,771) = 2.42, *p* = 0.010, partial η^2^ = 0.0028. Across both sexes, BMI category explained more variance in PFI than academic grade, particularly among males. In contrast, the BMI × grade interaction in males was statistically significant but very small in magnitude, indicating limited practical relevance despite the *p*-value reaching statistical significance.

### Nonlinear modeling of the BMI–PFI relationship

**Figure 2:**
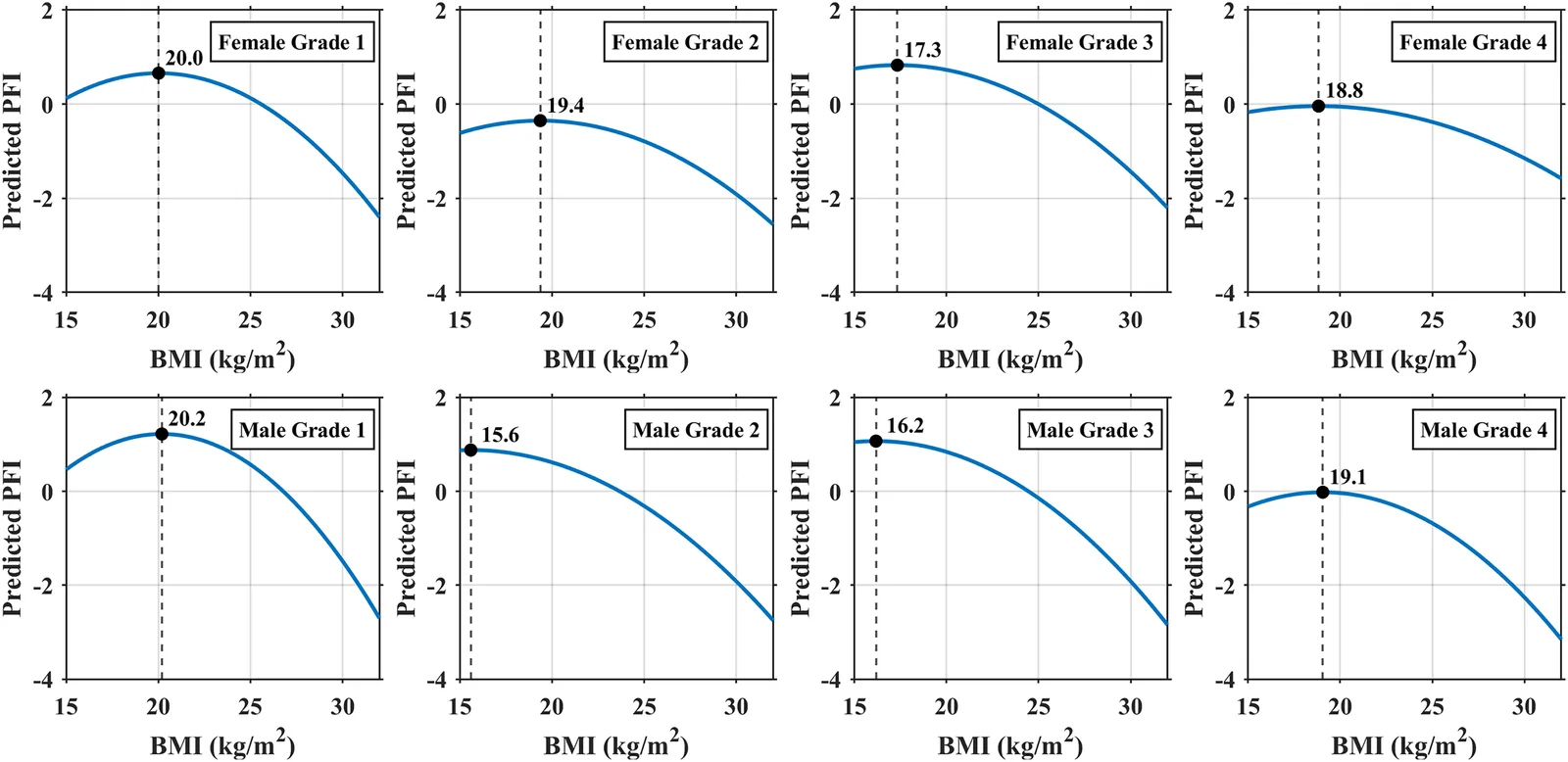
Sex- and grade-specific quadratic relationships between BMI and predicted PFI.

Figure 2 illustrates the quadratic relationships between BMI and predicted PFI across sex and grade. All eight models displayed a concave pattern, indicating that predicted PFI increased with BMI up to a model-derived peak and then declined at higher BMI values. Among females, predicted PFI reached its maximum at BMI values of approximately 20.0, 19.4, 17.3, and 18.8 for Grades 1–4, respectively. The curvature of the fitted models was moderate for Grades 1–3, whereas the Grade 4 curve was noticeably flatter, consistent with the smaller effect size observed in senior female students and suggesting a weaker dependence of PFI on BMI in this subgroup.

Male students demonstrated the same nonlinear trend but with steeper curves and greater separation across BMI categories. The model-derived BMI values associated with peak predicted PFI were approximately 20.2, 15.6, 16.2, and 19.1 for Grades 1–4, respectively. The lower peak estimates observed in Grades 2 and 3 fell below the conventional normal-weight range and therefore should not be interpreted as recommended BMI targets. Instead, they likely reflect the fitted model, the BMI distribution within these grade cohorts, and the relatively strong performance of lean male students in the observed sample. Beyond the peak, predicted PFI declined in the overweight and obese ranges, with more pronounced curvature observed in Grades 1–3.

Taken together, the nonlinear models show that the BMI–PFI relationship is consistently concave across sex and grade. The main interpretable finding is the presence of a nonlinear statistical pattern, particularly the decline in predicted PFI at higher BMI values, rather than the exact location of each estimated peak. Males exhibited steeper declines at higher BMI values, suggesting a more pronounced BMI–PFI association, whereas females showed a more moderate pattern, particularly in the senior grade.

### Correlation structure of fitness components

**Figure 3:**
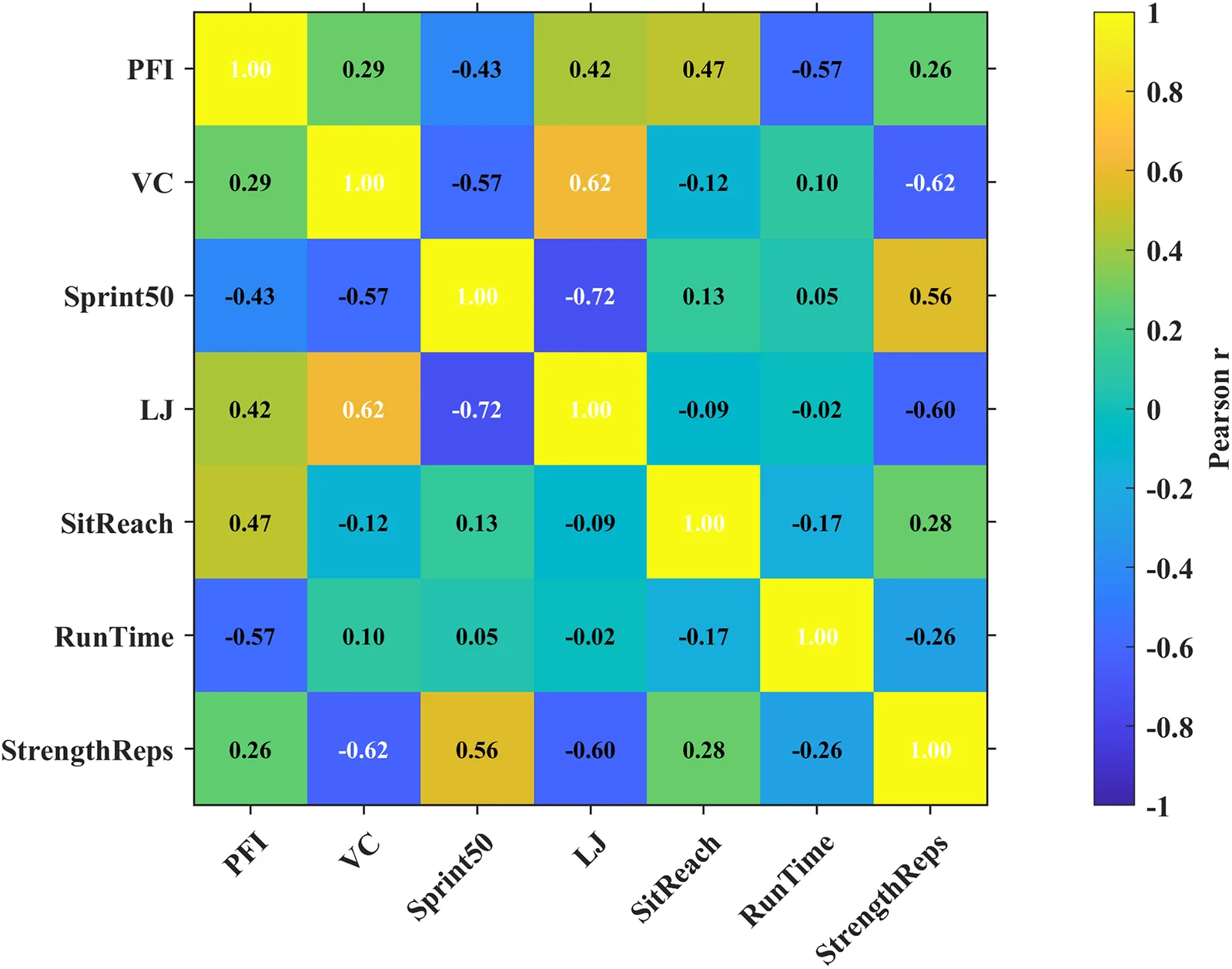
Correlation matrix of the physical fitness index (PFI) and individual fitness components.

The correlation matrix is shown in Fig. 3. PFI demonstrated moderate positive correlations with VC, LJ, sit-and-reach flexibility, and muscular endurance, indicating that higher performance in these components was associated with higher overall fitness scores. Negative correlations were found with both sprint time and endurance-run time, consistent with the inverse scoring direction of time-based variables.

Among individual components, lung capacity and long jump exhibited the strongest positive interrelationship (*r* ≈ 0.62), while sprint time showed strong negative associations with both (*r* = –0.43 for PFI and *r* = –0.72 for LJ). Flexibility displayed weaker correlations with most other variables, whereas endurance-run time was moderately associated with PFI (*r* = –0.57).

Overall, the observed correlation structure provided support for the use of PFI as a multidimensional composite index. The individual components were meaningfully associated with the overall PFI, but the pairwise correlations among components were not sufficiently high to indicate that any single test item was redundant or interchangeable with the others. This pattern is consistent with the intended role of the PFI as a surveillance-oriented index that summarizes several related but distinct fitness domains rather than a unidimensional scale.

Additional structure checks using the six sex-standardized and performance-oriented component scores showed no evidence of problematic multicollinearity. The maximum absolute pairwise correlation among the six components was 0.393, and VIF values ranged from 1.03 to 1.33. The KMO value was 0.711, and Bartlett’s test of sphericity was significant, χ^2^ (15) = 8,190.84, *p* < 0.001, indicating that the component correlation matrix was suitable for exploratory structure analysis. PCA identified two components with eigenvalues >1.0. The first component explained 33.1% of the variance, and the second explained 17.0%, together accounting for 50.1% of the total variance. These results suggest that the six components shared a meaningful general fitness dimension while also retaining domain-specific variation. Therefore, the PFI should be interpreted as a multidimensional unit-weighted surveillance index rather than as a unidimensional latent factor score.

## Discussion

The present study examined sex- and grade-related differences in physical fitness, BMI-related differences in PFI, and the nonlinear BMI–PFI relationship in a large university cohort. Four main findings emerged. First, marked sex differences were observed in individual fitness components, whereas the sex-standardized PFI showed no overall main effect of sex; grade-related variation was statistically significant but small in magnitude. Second, BMI category was associated with PFI, with normal-weight students generally showing higher PFI than underweight, overweight, and obese peers. Third, BMI and PFI followed a concave statistical pattern across sex–grade subgroups, with predicted PFI tending to be lower at both low and high BMI values. Finally, the correlation structure among fitness components provided support for the use of PFI as a multidimensional composite index. Together, these findings suggest that BMI status was more closely associated with multidimensional fitness than academic grade in this cohort, while sex-related differences were more evident in individual fitness components than in the sex-standardized composite score. Because of the cross-sectional design, these patterns should be interpreted as observed associations rather than evidence of directional effects between BMI and physical fitness.

### Sex- and grade-related differences in physical fitness

Consistent with well-established physiological distinctions after puberty, males outperformed females in vital capacity, jumping performance, sprint speed, muscular endurance, and distance running, with only small sex differences in flexibility. These results align with evidence that males possess greater skeletal muscle mass, larger type II fiber area, higher testosterone, and superior aerobic and anaerobic capacity, which collectively enhance strength, power, and running performance (Nuzzo, 2023; Hunter & Senefeld, 2024; Miller et al., 2024). Similar performance gaps have been reported consistently in university-aged samples (Martins et al., 2023; Solli, Sandbakk & McGawley, 2024).

Beyond component-level differences, the sex-standardized PFI showed no overall main effect of sex, whereas academic grade showed a significant but small main effect. The sex × grade interaction was also significant, indicating that the pattern of PFI variation across academic years differed slightly between males and females. Given the large sample size, some statistically significant findings corresponded to very small effect sizes. Therefore, the grade-related and sex × grade findings should be interpreted cautiously as modest population-level differences rather than strong practical effects. This pattern is not unexpected because the PFI was constructed from sex-standardized component scores, which reduces between-sex differences in the overall composite metric. The association between academic grade and PFI may reflect differences in lifestyle-related factors across university years, such as physical activity, sleep patterns, academic workload, and nutrition, rather than age-related physiological change alone (Perez-Gomez et al., 2008; Deliens et al., 2015).

The significant sex × grade interaction indicates that fitness trajectories differed between males and females. In males, PFI in Grades 2–4 was slightly lower than in Grade 1, fluctuating around zero without a monotonic decline. In females, PFI was lowest in Grade 2 but comparable across Grades 1, 3, and 4. These modest and non-linear shifts may reflect sex-specific behavioral or lifestyle differences during university rather than age-related physiological change alone. Prior research shows that males often experience sharper increases in sedentary activity, greater weight gain, and larger reductions in habitual physical activity across university years, which may be associated with lower integrated fitness indicators such as PFI (Deliens et al., 2015; Górski et al., 2018). Although females often report lower physical activity on average, their weight-related and movement behaviors may vary differently across academic years, which could contribute to the observed sex-specific pattern (Beville et al., 2014; McCarthy & Warne, 2022).

The observed grade-related fluctuations therefore likely reflect behavioral and environmental shifts rather than age-driven physiology. Supporting this view, multiple studies show that strength, endurance, and body composition in university students are highly sensitive to changes in exercise frequency, training intensity, and overall activity—even in the absence of major biological maturation (Kapsis et al., 2022; Solli, Sandbakk & McGawley, 2024). Flexibility exhibited substantial variability without a consistent grade pattern, consistent with findings that sit-and-reach performance is influenced more by posture, stretching, and lifestyle than by chronological age (Martins et al., 2023; Nuzzo, 2023).

Overall, multidimensional fitness in university students appeared to be more closely associated with BMI status than with academic grade, whereas sex-specific patterns were more evident at the level of individual fitness components than in the sex-standardized composite PFI. These findings suggest that sex-specific interpretation may be useful when evaluating fitness profiles or designing interventions.

### BMI-related differences in physical fitness index

BMI category was associated with PFI in this cohort, with the normal-weight group generally showing the highest PFI and the overweight and obese groups showing lower values. This pattern is consistent with previous large-scale studies reporting lower health-related fitness among students outside the normal BMI range (Ding & Jiang, 2020; Li et al., 2023; Wu et al., 2024). However, these findings should not be interpreted as indicating that lower BMI is uniformly beneficial. Rather, the BMI–PFI association may reflect the balance between body mass, body composition, relative strength, and the mechanical and metabolic demands of the fitness tests.

The lower PFI observed in overweight and obese students may be partly explained by the additional non-functional mass that must be accelerated, lifted, and transported during weight-bearing tasks such as sprinting, standing long jump, sit-ups, and middle-distance running. Excess body mass may reduce relative power output, increase the energetic cost of locomotion, and place greater mechanical demand on the lower limbs. These mechanisms are consistent with evidence that overweight and obese youth often perform worse in running, jumping, and muscular endurance tests, and that higher body mass is associated with altered gait biomechanics and greater lower-limb loading during locomotor tasks (Deforche et al., 2003; Mak et al., 2010; Ruiz et al., 2011; Ortega, Ruiz & Castillo, 2013; Molina-Garcia et al., 2019).

The finding that underweight students performed better than overweight and obese students in several comparisons requires careful interpretation. In weight-bearing field tests, lower body mass may reduce the mechanical load that must be moved during running, jumping, and trunk-flexion tasks. This may partly explain why underweight students outperformed overweight and obese groups in some test combinations. Nevertheless, this does not imply that underweight status is physiologically advantageous. Low BMI may reflect lower fat-free mass, reduced muscle cross-sectional area, or insufficient nutritional reserve, which can limit force production, repeated-effort capacity, and endurance performance. Previous research has shown that underweight status, and not only overweight or obesity, can be associated with poorer health-related fitness compared with normal weight (Artero et al., 2010; Mak et al., 2010; Kung et al., 2020).

The stronger BMI-category effect observed in males may reflect sex-related differences in body size, fat distribution, and relative strength during weight-bearing performance tests. Because males generally have higher absolute body mass and may accumulate central adiposity more readily during early adulthood, excess BMI may impose greater locomotor and mechanical penalties during running and jumping tasks. This may help explain why the BMI–PFI association was more pronounced in males than in females. However, because body composition was not directly measured, this explanation should be interpreted as a plausible mechanism rather than a confirmed causal pathway.

When BMI category and academic grade were considered simultaneously within each sex, BMI category explained more variance in PFI than academic grade. The BMI × grade interaction was not significant in females and was statistically significant but very small in males. Therefore, BMI-related differences in PFI appeared broadly consistent across academic grades, whereas grade-related differences were comparatively modest. This interpretation is consistent with the effect-size pattern observed in the present study and avoids treating statistically significant but very small interaction effects as practically large findings.

### Nonlinear BMI–PFI relationship

Quadratic models demonstrated a consistent concave BMI–PFI pattern across all sex–grade subgroups. The estimated peak values represented statistical vertices from the fitted models rather than physiological optima. This distinction is important because several peak estimates, especially in male Grades 2 and 3, fell below the conventional normal-weight range. Therefore, these estimates should be interpreted as model-dependent descriptions of the observed data rather than as recommended BMI targets or evidence that underweight status is advantageous.

Similar nonlinear associations have been reported across youth and university populations. Chinese school-aged children and Tibetan adolescents have shown higher PFI near lower-to-normal BMI values, with declines toward both ends of the BMI continuum (Zhao et al., 2012; Guo et al., 2024). Large population-based studies likewise show curvilinear relationships between BMI and health-related fitness, with stronger nonlinear patterns in boys than girls (Fogelholm et al., 2008; Chen et al., 2022). Recent findings in Chinese university cohorts confirm that underweight, overweight, and obese students score lower on PFI and that nonlinear models may describe BMI–PFI associations more appropriately than purely linear models (Lu et al., 2014; Li et al., 2022; Wu et al., 2024).

The mechanisms underlying this concave pattern may differ at the lower and upper BMI extremes. At the lower end, underweight students may have lower fat-free mass, reduced force-generating capacity, and limited nutritional reserves, which can constrain explosive power, muscular endurance, and running performance. At the higher end, overweight and obese students—particularly males—may experience greater mechanical loading, reduced relative strength, and higher energetic cost during running, jumping, and repeated trunk-flexion tasks. These mechanisms are consistent with previous evidence linking low BMI with reduced muscular fitness and higher BMI with poorer locomotor and weight-bearing performance (Artero et al., 2010; Mak et al., 2010; Kwieciński et al., 2018; Bi et al., 2019; Molina-Garcia et al., 2019).

Although the quadratic model provided an interpretable summary of the overall BMI–PFI pattern, it cannot capture all possible local variations in the BMI–fitness relationship. Future studies should compare quadratic models with more flexible approaches, such as restricted cubic splines or generalized additive models, particularly in longitudinal or multi-cohort datasets.

The sex-specific curves observed here are consistent with previous findings that BMI–fitness associations are often more pronounced in males (Chen et al., 2022; Wu et al., 2024; Li, Shi & Tao, 2025). Higher absolute body mass and central adiposity may exacerbate locomotor and metabolic penalties in males, contributing to steeper declines in predicted PFI at higher BMI values. In contrast, the flatter curves in senior females may reflect greater heterogeneity in lifestyle, training habits, or daily activity, which could weaken the apparent BMI–PFI association in this subgroup.

From a practical perspective, these nonlinear patterns suggest that university fitness monitoring should consider both low and high BMI as potential markers of lower multidimensional fitness, rather than focusing only on weight reduction. This is consistent with population-specific BMI considerations for Chinese college students that emphasize avoiding both underweight and obesity (Wang et al., 2022). Accordingly, interventions should aim to improve body composition and functional performance simultaneously, particularly in male students, among whom the BMI–PFI association appeared more pronounced.

### Correlation structure of fitness components

In the present study, the Physical Fitness Index (PFI) showed moderate positive correlations with vital capacity (*r* ≈ 0.29), standing long jump (*r* ≈ 0.42), sit-and-reach flexibility (*r* ≈ 0.47), and muscular endurance (StrengthReps, *r* ≈ 0.26), alongside moderate negative correlations with 50-m sprint time (*r* ≈ −0.43) and middle-distance running time (*r* ≈ −0.57). This configuration indicates that higher PFI scores reflect concurrent advantages in cardiorespiratory function, lower-limb power, flexibility, and muscular endurance, as well as superior sprint and endurance-run performance. The multicomponent nature of these associations supports the interpretation that PFI synthesizes diverse physiological domains rather than privileging any single fitness component.

Comparable correlation structures have been consistently reported in field-based batteries for youth and young adults. The ALPHA health-related fitness test battery, for example, demonstrated that cardiorespiratory fitness, lower-limb power, and speed–agility cluster together and exhibit moderate-to-strong mutual correlations, supporting their integration into composite indices (Ruiz et al., 2011). Similarly, Chinese school-based surveillance systems have shown that vital capacity, standing long jump, sprint performance, flexibility, and distance-run time form a coherent health-related fitness construct, with running times inversely related to VC and power-based tests and positively related to overall fitness grading (Qin, Qin & Liu, 2022). Studies in Chinese university students have also confirmed that distance-run performance and long-jump power contribute substantially to aggregated fitness classifications (Dong et al., 2023; Guo, 2025).

Within the present dataset, the strongest bivariate associations were found between standing long jump and sprint time (*r* ≈ −0.72) and between standing long jump and muscular endurance (*r* ≈ −0.60), while VC showed moderate correlations with both sprint and long-jump performance. These patterns align with evidence that neuromuscular and biomechanical determinants—such as lower-limb force production, rate of force development, and elastic energy utilization—underlie both explosive power and running speed, leading to co-variation across these tests (Ruiz et al., 2011; Ramírez-Vélez et al., 2015). Importantly, all correlations were well below unity, indicating that each component contributes unique variance to the PFI and that no single test can serve as a proxy for multidimensional fitness.

Flexibility demonstrated only modest correlations with other components (|*r*| generally <0.30), a pattern reported in multiple large-scale analyses in which sit-and-reach loads weakly onto primary cardiorespiratory–muscular fitness factors (Ruiz et al., 2011; Ramírez-Vélez et al., 2015). This suggests that flexibility represents a distinct physiological attribute and contributes additional information to the PFI without overlapping substantially with power or endurance constructs.

Collectively, the observed correlation structure supports the use of PFI as a practical multicomponent summary index: cardiorespiratory capacity and lower-limb power emerged as central contributors, while flexibility and muscular endurance provided partly independent information on students’ functional capacity. The additional VIF and PCA results further support this interpretation. The low VIF values indicate that the six components were not highly collinear, while the PCA results suggest that the component set contained both a general fitness dimension and domain-specific variation. This pattern is consistent with the intended purpose of the PFI as a multidimensional surveillance index rather than a unidimensional latent construct.

The relatively weak association between flexibility and the other fitness components should therefore not be interpreted as a limitation of the PFI. Sit-and-reach performance reflects joint range of motion, hamstring and lower-back flexibility, test posture, and stretching-related behavior, which are partly distinct from the neuromuscular and cardiorespiratory determinants of running, jumping, and muscular endurance. Its weaker correlations indicate that flexibility contributes additional information to the composite index rather than duplicating the information provided by power or endurance tests.

These findings may have practical relevance for fitness assessment and intervention design, provided that they are interpreted within the limits of the cross-sectional study design. Programs emphasizing only endurance running may not produce broad improvements in PFI unless they also target strength, power, and flexibility components that jointly shape integrated fitness profiles.

### Practical implications

The findings provide several cautious implications for university fitness monitoring and health promotion. First, the observed BMI–PFI association suggests that BMI may be used as a preliminary screening indicator, but it should not be treated as a direct measure of body composition or physical fitness. BMI-based screening may help identify students who could benefit from follow-up fitness assessment, body-composition evaluation when available, and individualized guidance on physical activity and weight management. Second, the correlation and exploratory structure analyses support the interpretation of PFI as a multidimensional surveillance index that integrates cardiorespiratory function, lower-limb power, flexibility, muscular endurance, and running performance. Training programs designed to improve overall fitness should therefore adopt balanced approaches incorporating endurance, strength, power, and flexibility training rather than relying solely on long-distance running. Third, the modest grade-related differences suggest that academic year alone is unlikely to provide sufficient information for identifying students with lower physical fitness. University health-promotion strategies may therefore benefit from combining BMI status, sex-specific fitness profiles, and multidimensional fitness testing when designing physical education curricula and targeted intervention programs.

### Limitations and future directions

Several limitations warrant consideration. The cross-sectional design precludes causal inference regarding the directionality of BMI–PFI associations or the temporal ordering between weight status and physical fitness. Longitudinal studies are needed to determine how within-person changes in weight status relate to fitness trajectories. BMI was the sole index of weight status and does not differentiate between fat and lean mass. This limitation is particularly relevant in university populations because students with greater fat-free mass or regular sport participation may be classified as overweight by BMI despite having a relatively favorable body-composition profile. Conversely, students with normal BMI may still have low muscle mass or higher adiposity. Therefore, BMI-category differences in PFI should be interpreted as weight-status associations rather than direct evidence of adiposity- or muscle-mass-related effects. Incorporating direct assessments of body composition—such as bioelectrical impedance, dual-energy X-ray absorptiometry (DXA), or skinfold measurement—would allow future studies to distinguish fat mass, fat-free mass, and muscle-related profiles, thereby improving interpretation of the nonlinear BMI–PFI patterns. Participant inclusion was based on the availability of complete de-identified institutional fitness records. Because the dataset did not contain health-screening information or detailed information on students with incomplete or invalid records, it was not possible to determine whether missing or invalid records were related to medical conditions, musculoskeletal injuries, or other factors that could impair field-test performance. Therefore, potential selection bias cannot be fully excluded, and the direction or magnitude of this bias could not be estimated. In addition, the dataset did not contain information on medical history, recent musculoskeletal injury, habitual physical activity, sedentary behavior, dietary patterns, sleep quality, or training status. These behavioral and health-related variables may influence both BMI and physical fitness and therefore represent important unmeasured confounders in the observed BMI–PFI associations. Because these variables were unavailable, the present analyses could not estimate the independent association between BMI and PFI after adjustment for lifestyle, training, injury, or health-status factors. Therefore, residual confounding should be considered when interpreting the findings.

Although the PFI provided a practical summary of multidimensional physical fitness, it was constructed as a unit-weighted composite index and has not been externally validated against independent health outcomes or direct physiological measures. Exploratory PCA was used only to describe the internal structure of the six fitness components and should not be regarded as validation of the PFI. Future studies should examine the criterion validity and longitudinal responsiveness of the PFI and compare the present unit-weighted approach with alternative weighting strategies.

The nonlinear analysis was based on quadratic models to provide an interpretable single-peak summary of the BMI–PFI association. This approach was useful for describing the overall concave pattern but may not capture all local nonlinear variations across the BMI continuum. Future studies should compare quadratic models with more flexible approaches, such as restricted cubic splines or generalized additive models, particularly in longitudinal or multi-cohort datasets.

Finally, the sample was drawn from a specific university population and regional testing context, and the findings may not generalize to students from other universities, regions, countries, age groups, or educational systems. Even within China, university students may differ in socioeconomic background, admission characteristics, physical education requirements, campus sport resources, regional lifestyle patterns, and testing implementation procedures. Because the data were obtained from a standardized Chinese university fitness-testing program, replication in multi-campus and demographically diverse cohorts is necessary before applying these findings to broader student populations.

## Conclusions

This study characterized sex-, grade-, and BMI-related patterns in multidimensional physical fitness among university students. Sex differences were evident across individual fitness components, whereas the sex-standardized composite PFI showed no overall main effect of sex and only modest grade-related variation. BMI was associated with PFI, with normal-weight students generally showing higher values and both underweight and overweight/obese groups demonstrating lower performance. Quadratic models further indicated a concave BMI–PFI pattern across sex–grade subgroups. The model-derived peak estimates varied across subgroups and should be interpreted as statistical features of the fitted models rather than physiological optima or recommended BMI targets. Therefore, the principal nonlinear finding is the decline in predicted PFI at higher BMI values, rather than a universal BMI range associated with peak fitness. The correlation structure provided support for the interpretation that PFI captured contributions from several distinct yet interrelated fitness domains.

Overall, these findings suggest that weight status was more closely associated with multidimensional fitness than academic grade in this cohort. BMI may be useful as a preliminary screening indicator in university fitness monitoring, but it should not be treated as a direct measure of body composition or physical fitness. The results may inform BMI-informed and sex-sensitive health-promotion strategies in university settings when combined with multidimensional fitness testing and, where possible, body-composition and behavioral assessment. Because this study used a cross-sectional design and did not include direct body-composition, behavioral, physiological, or biomechanical measures, the findings should be interpreted as associations within the observed cohort rather than evidence of causal relationships. In addition, because the sample was drawn from a specific university population and regional testing context in China, the findings should be generalized cautiously to students from other universities, regions, or educational systems. Future longitudinal and multi-campus studies incorporating direct body-composition measures, behavioral indicators, and physiological assessments are needed to better understand fitness development across the university years.

## Supplemental Information

10.7717/peerj.21656/supp-1 Supplemental Information 1 STROBE Checklist.

10.7717/peerj.21656/supp-2 Supplemental Information 2 Raw data.

10.7717/peerj.21656/supp-3 Supplemental Information 3 English Codebook for categorical variables.

## Additional Information and Declarations

### Data Availability

The following information was supplied regarding data availability:

The data is available in the Supplemental files.

## References

[ref-53] Artero EG, España-Romero V, Ortega FB, Jiménez-Pavón D, Ruiz JR, Vicente-Rodríguez G, Bueno M, Marcos A, Gómez-Martínez S, Urzanqui A, González-Gross M, Moreno LA, Gutiérrez A, Castillo MJ (2010). Health-related fitness in adolescents: underweight, and not only overweight, as an influencing factor. The AVENA study. Scandinavian Journal of Medicine & Science in Sports.

[ref-1] Beville JM, Umstattd Meyer MR, Usdan SL, Turner LW, Jackson JC, Lian BE (2014). Gender differences in college leisure time physical activity: application of the theory of planned behavior and integrated behavioral model. Journal of American College Health.

[ref-2] Bi C, Yang J, Sun J, Song Y, Wu X, Zhang F (2019). Benefits of normal body mass index on physical fitness: a cross-sectional study among children and adolescents in Xinjiang Uyghur Autonomous Region, China. PLOS ONE.

[ref-3] Carnethon MR, Gidding SS, Nehgme R, Sidney S, Jacobs DR, Liu K (2003). Cardiorespiratory fitness in young adulthood and the development of cardiovascular disease risk factors. The Journal of the American Medical Association.

[ref-4] Chen G, Chen J, Liu J, Hu Y, Liu Y (2022). Relationship between body mass index and physical fitness of children and adolescents in Xinjiang, China: a cross-sectional study. BMC Public Health.

[ref-5] Deforche B, Lefevre J, De Bourdeaudhuij I, Hills AP, Duquet W, Bouckaert J (2003). Physical fitness and physical activity in obese and nonobese Flemish youth. Obesity Research.

[ref-6] Deliens T, Deforche B, De Bourdeaudhuij I, Clarys P (2015). Changes in weight, body composition and physical fitness after 1.5 years at university. European Journal of Clinical Nutrition.

[ref-7] Ding C, Jiang Y (2020). The relationship between body mass index and physical fitness among Chinese university students: results of a longitudinal study. Healthcare.

[ref-8] Dong X, Huang F, Starratt G, Yang Z (2023). Trend in health-related physical fitness for Chinese male first-year college students: 2013–2019. Frontiers in Public Health.

[ref-9] Fogelholm M, Stigman S, Huisman T, Metsämuuronen J (2008). Physical fitness in adolescents with normal weight and overweight. Scandinavian Journal of Medicine & Science in Sports.

[ref-11] Gómez-Campos R, Vidal-Espinoza R, Lazari E, Urra-Albornoz C, de Campos LFCC, Rivera-Portugal M, Cossio-Bolaños M (2025). Relationship between body mass index and lower limb power in children and adolescents. European Journal of Translational Myology.

[ref-12] Górski M, Starczewski M, Pastuszak A, Mazur-Różycka J, Gajewski J, Buśko K (2018). Changes of strength and maximum power of lower extremities in adolescent handball players during a two-year training cycle. Journal of Human Kinetics.

[ref-13] Guo H (2025). Risk identification and improvement strategies for BMI and physical fitness and health grade cross-classification: a cross-sectional study based on Chinese college students. Frontiers in Public Health.

[ref-14] Guo T, Shen S, Yang S, Yang F (2024). The relationship between BMI and physical fitness among 7451 college freshmen: a cross-sectional study in Beijing, China. Frontiers in Physiology.

[ref-15] Guthold R, Stevens GA, Riley LM, Bull FC (2018). Worldwide trends in insufficient physical activity from 2001 to 2016: a pooled analysis of 358 population-based surveys with 1.9 million participants. The Lancet Global Health.

[ref-16] Haase A, Steptoe A, Sallis JF, Wardle J (2004). Leisure-time physical activity in university students from 23 countries: associations with health beliefs, risk awareness, and national economic development. Preventive Medicine.

[ref-17] Hunter SK, Senefeld JW (2024). Sex differences in human performance. The Journal of Physiology.

[ref-18] Joyner MJ, Hunter SK, Senefeld JW (2025). Evidence on sex differences in sports performance. Journal of Applied Physiology.

[ref-19] Kapsis DP, Tsoukos A, Psarraki MP, Douda HT, Smilios I, Bogdanis GC (2022). Changes in body composition and strength after 12 weeks of high-intensity functional training with two different loads in physically active men and women: a randomized controlled study. Sports.

[ref-20] Kung Y-T, Chang C-M, Hwang F-M, Chi S-C (2020). The association between body mass index and physical fitness of normal weight/overweight/obese university students. International Journal of Environmental Research and Public Health.

[ref-21] Kwieciński J, Konarski JM, Strzelczyk R, Krzykała M, Konarska A, Bartkowiak S, Lopes V, Malina RM (2018). Non-linear relationships between the BMI and physical fitness in Polish adolescents. Annals of Human Biology.

[ref-24] Li S, Cao H, Liu H, Hu Y, Liu J (2023). Relationship between body mass index and physical fitness index in Chinese college students: results from a cross-sectional survey. American Journal of Human Biology.

[ref-25] Li Y, Shi N, Tao Y (2025). Associations between body mass index and health-related physical fitness among Chinese university students: a cross-sectional study. Frontiers in Sports and Active Living.

[ref-26] Li B, Sun L, Yu Y, Xin H, Zhang H, Liu J, Zhang Z (2022). Associations between body composition and physical fitness among Chinese medical students: a cross-sectional study. BMC Public Health.

[ref-27] Lopes VP, Cossio-Bolaños M, Gómez-Campos R, De Arruda M, Hespanhol JE, Rodrigues LP (2017). Linear and nonlinear relationships between body mass index and physical fitness in Brazilian children and adolescents. American Journal of Human Biology.

[ref-28] Lu Y-J, Zheng X-D, Zhou F-S, Zuo X-B (2014). BMI and physical fitness in Chinese adult students: a large school-based analysis. International Journal of Clinical and Experimental Medicine.

[ref-51] Mak KK, Ho SY, Lo WS, Thomas GN, McManus AM, Day JR, Lam TH (2010). Health-related physical fitness and weight status in Hong Kong adolescents. BMC Public Health.

[ref-29] Martins HA, Barbosa JG, Seffrin A, Vivan L, Souza VRDA, De Lira CAB, Weiss K, Knechtle B, Andrade MS (2023). Sex differences in maximal oxygen uptake adjusted for skeletal muscle mass in amateur endurance athletes: a cross sectional study. Healthcare.

[ref-31] McCarthy C, Warne JP (2022). Gender differences in physical activity status and knowledge of Irish University staff and students. Sport Sciences for Health.

[ref-33] Miller R, Balshaw TG, Massey GJ, Maeo S, Lanza MB, Haug B, Johnston M, Allen SJ, Folland JP (2024). Sex differences in muscle morphology between male and female sprinters. Journal of Applied Physiology.

[ref-52] Molina-Garcia P, Migueles JH, Cadenas-Sanchez C, Esteban-Cornejo I, Mora-Gonzalez J, Rodriguez-Ayllon M, Plaza-Florido A, Molina-Molina A, Garcia-Delgado G, D’Hondt E, Vanrenterghem J, Ortega FB (2019). Fatness and fitness in relation to functional movement quality in overweight and obese children. Journal of Sports Sciences.

[ref-34] Nuzzo JL (2023). Narrative review of sex differences in muscle strength, endurance, activation, size, fiber type, and strength training participation rates, preferences, motivations, injuries, and neuromuscular adaptations. The Journal of Strength & Conditioning Research.

[ref-35] Ortega FB, Ruiz JR, Castillo MJ (2013). Physical activity, physical fitness, and overweight in children and adolescents: evidence from epidemiologic studies. Endocrinología y Nutrición (English Edition).

[ref-36] Ortega FB, Ruiz JR, Castillo MJ, Sjöström M (2008). Physical fitness in childhood and adolescence: a powerful marker of health. International Journal of Obesity.

[ref-37] Perez-Gomez J, Rodriguez GV, Ara I, Olmedillas H, Chavarren J, González-Henriquez JJ, Dorado C, Calbet JAL (2008). Role of muscle mass on sprint performance: gender differences?. European Journal of Applied Physiology.

[ref-38] Qin G, Qin Y, Liu B (2022). Association between BMI and health-related physical fitness: a cross-sectional study in Chinese high school students. Frontiers in Public Health.

[ref-39] Ramírez-Vélez R, Rodrigues-Bezerra D, Correa-Bautista JE, Izquierdo M, Lobelo F (2015). Reliability of health-related physical fitness tests among Colombian children and adolescents: the FUPRECOL study. PLOS ONE.

[ref-40] Ruiz JR, Castro-Piñero J, España-Romero V, Artero EG, Ortega FB, Cuenca MM, Jimenez-Pavón D, Chillón P, Girela-Rejón MJ, Mora J, Gutiérrez Á, Suni J, Sjöström M, Castillo MJ (2011). Field-based fitness assessment in young people: the ALPHA health-related fitness test battery for children and adolescents. British Journal of Sports Medicine.

[ref-43] Solli GS, Sandbakk Ø, McGawley K (2024). Sex differences in performance and performance-determining factors in the Olympic winter endurance sports. Sports Medicine—Open.

[ref-50] Sun F, He Q, Sun X, Wang J (2022). The association between body mass index and muscular fitness in chinese college freshmen. International Journal of Environmental Research and Public Health.

[ref-44] Tomkinson GR, Lang JJ, Tremblay MS, Dale M, LeBlanc AG, Belanger K, Ortega FB, Léger L (2017). International normative 20 m shuttle run values from 1,142,026 children and youth representing 50 countries. British Journal of Sports Medicine.

[ref-45] Wang Z, Wang J, Shi Y, Fang Q, Tan Q, Wang M, Li J (2022). Optimal BMI cutoff points in obesity screening for Chinese college students. Frontiers in Psychology.

[ref-46] Wu H, Lin P, Zeng G, Chen F (2024). Associations between body mass index and physical fitness indicators among Chinese university students: a multicenter cross-sectional study. BMC Sports Science, Medicine and Rehabilitation.

[ref-47] Zhao Y, Wang F, Zhu P, Liu R, Hao J, Su P, Wang L, Zu P, Tao F-B (2012). Association between body mass index and physical fitness index among children and adolescents. Zhonghua liu Xing Bing xue za zhi = Zhonghua Liuxingbingxue Zazhi.

