# Supplementary material for: Body mass index and physical fitness in university students: sex differences and nonlinear patterns in a large cross-sectional study
Source: PeerJ. 2026 Aug 11;14:e21656. doi: 10.7717/peerj.21656 (PMC13475385; doi:10.7717/peerj.21656)
Supplement: Supplementary file 1 [file peerj-14-21656-s003.docx]

**Codebook for the De-identified Dataset**

*Body mass index and physical fitness in university students: sex differences and nonlinear patterns in a large cross-sectional study*

**Description.** This file describes the variables included in the supplementary dataset. All personal identifiers were removed prior to submission. Categorical variables are presented as English text labels for clarity. Blank cells indicate that the variable was not applicable to that participant under the sex-specific physical fitness testing protocol.

| **Variable name** | **Description** | **Coding / unit** |
| --- | --- | --- |
| **Grade** | Academic grade | Grade 1, Grade 2, Grade 3, Grade 4 |
| **Sex** | Biological sex | Female, Male |
| **Height_cm** | Body height | cm |
| **Weight_kg** | Body weight | kg |
| **Weight_score** | Standardized score for body weight/BMI based on the national physical fitness assessment system | points |
| **BMI_category** | BMI classification | Underweight, Normal, Overweight, Obese |
| **Vital_capacity_ml** | Vital capacity | mL |
| **Vital_capacity_score** | Standardized score for vital capacity | points |
| **Vital_capacity_level** | Performance category for vital capacity | Excellent, Good, Pass, Fail |
| **Sprint_50m_s** | 50-m sprint time | s |
| **Sprint_50m_score** | Standardized score for 50-m sprint | points |
| **Sprint_50m_level** | Performance category for 50-m sprint | Excellent, Good, Pass, Fail |
| **Standing_long_jump_cm** | Standing long jump distance | cm |
| **Standing_long_jump_score** | Standardized score for standing long jump | points |
| **Standing_long_jump_level** | Performance category for standing long jump | Excellent, Good, Pass, Fail |
| **Sit_and_reach_cm** | Sit-and-reach distance | cm |
| **Sit_and_reach_score** | Standardized score for sit-and-reach | points |
| **Sit_and_reach_level** | Performance category for sit-and-reach | Excellent, Good, Pass, Fail |
| **Run_800m_min_sec** | 800-m run time | females only; recorded in minute–second format, e.g., 4′54 = 4 min 54 s |
| **Run_800m_score** | Standardized score for 800-m run | females only, points |
| **Run_800m_level** | Performance category for 800-m run | females only: Excellent, Good, Pass, Fail |
| **Run_800m_bonus** | Bonus score for 800-m run | females only, points |
| **Run_1000m_min_sec** | 1000-m run time | males only; recorded in minute–second format, e.g., 5′01 = 5 min 01 s |
| **Run_1000m_score** | Standardized score for 1000-m run | males only, points |
| **Run_1000m_level** | Performance category for 1000-m run | males only: Excellent, Good, Pass, Fail |
| **Run_1000m_bonus** | Bonus score for 1000-m run | males only, points |
| **Sit_ups_1min_reps** | One-minute sit-ups | females only, repetitions |
| **Sit_ups_1min_score** | Standardized score for one-minute sit-ups | females only, points |
| **Sit_ups_1min_level** | Performance category for one-minute sit-ups | females only: Excellent, Good, Pass, Fail |
| **Sit_ups_1min_bonus** | Bonus score for one-minute sit-ups | females only, points |
| **Pull_ups_reps** | Pull-ups | males only, repetitions |
| **Pull_ups_score** | Standardized score for pull-ups | males only, points |
| **Pull_ups_level** | Performance category for pull-ups | males only: Excellent, Good, Pass, Fail |
| **Pull_ups_bonus** | Bonus score for pull-ups | males only, points |

**Notes**

1. The dataset was derived from routine university physical fitness testing records.
2. All personal identifiers were removed before submission.
3. Categorical variables are reported as English text labels rather than numeric codes to improve interpretability for editors, reviewers, and readers.
4. Blank cells indicate that the corresponding test item was not applicable to that participant because of sex-specific assessment protocols.
5. The score variables represent values assigned according to the national standardized physical fitness assessment system used in China.
6. Performance categories were translated as: Excellent, Good, Pass, and Fail.
7. BMI categories were translated as: Underweight, Normal, Overweight, and Obese.
