# Supplementary material for: Body mass index and physical fitness in university students: sex differences and nonlinear patterns in a large cross-sectional study
Source: PeerJ. 2026 Aug 11;14:e21656. doi: 10.7717/peerj.21656 (PMC13475385; doi:10.7717/peerj.21656)
Supplement: Supplementary file 2 [file peerj-14-21656-s001.doc]

STROBE Statement—Checklist of items that should be included in reports of ***cross-sectional studies***

|  | Item No | Recommendation |
| --- | --- | --- |
| **Title and abstract** | 1 | (*a*) Indicate the study’s design with a commonly used term in the title or the abstract.  Reported in the Abstract (Methods), where the study is described as a cross-sectional study. |
|  |  | (*b*) Provide in the abstract an informative and balanced summary of what was done and what was found.  Reported in the structured Abstract (Background, Methods, Results, Conclusion), which summarizes the study background, sample size, cross-sectional design, PFI construction, statistical analyses, main findings, and cautious interpretation of the results. |
| Introduction | | |
| Background/rationale | 2 | Explain the scientific background and rationale for the investigation being reported.  Reported in the Introduction, which describes previous evidence on BMI and physical fitness, the limitations of linear and categorical BMI analyses, the rationale for using a multidimensional Physical Fitness Index, and the need to examine sex- and grade-specific nonlinear BMI–PFI patterns. |
| Objectives | 3 | State specific objectives, including any prespecified hypotheses.  Reported in the final paragraph of the Introduction, which states the objectives to examine sex- and grade-related differences in multidimensional physical fitness, compare PFI across BMI categories within each sex, and characterize nonlinear BMI–PFI relationships using sex- and grade-specific quadratic models. |
| Methods | | |
| Study design | 4 | Present key elements of study design early in the paper.  Reported in the Abstract (Methods) and Materials & Methods, where the study is described as a cross-sectional analysis of de-identified institutional fitness-testing records. |
| Setting | 5 | Describe the setting, locations, and relevant dates, including periods of recruitment, exposure, follow-up, and data collection.  Reported in Materials & Methods (Ethics approval and informed consent; Participants), describing the university setting, routine annual physical fitness assessment, institutional data source, and ethical approval information. Follow-up was not applicable because this was a cross-sectional study. |
| Participants | 6 | (*a*) Give the eligibility criteria, and the sources and methods of selection of participants  Reported in Materials & Methods (Participants), stating that participants were selected from de-identified institutional records of students who completed the standardized annual physical fitness assessment and had complete anthropometric and six-component fitness-test data.  (*b*) For matched studies, give matching criteria and number of exposed and unexposed.  Not applicable. This was not a matched study. |
| Variables | 7 | Clearly define all outcomes, exposures, predictors, potential confounders, and effect modifiers. Give diagnostic criteria, if applicable  Reported in Materials & Methods (Anthropometric Measures, Physical Fitness Assessment, Physical Fitness Index, Statistical Analysis). BMI, BMI categories, sex, academic grade, individual fitness components, and PFI are defined. The manuscript also states that health-related and behavioral variables, including medical history, recent musculoskeletal injury, habitual physical activity, sedentary behavior, dietary intake, sleep quality, and training status, were not available and therefore could not be used as exclusion criteria or covariates. |
| Data sources/ measurement | 8* | For each variable of interest, give sources of data and details of methods of assessment (measurement). Describe comparability of assessment methods if there is more than one group  Reported in Materials & Methods (Anthropometric Measures; Physical Fitness Assessment; Physical Fitness Index). Height and body mass measurement, BMI calculation, BMI classification, standardized national fitness tests, sex-specific test items, z-score standardization, and reverse scoring of time-based tests are described. Testing was conducted using standardized protocols and calibrated equipment. |
| Bias | 9 | Describe any efforts to address potential sources of bias  Reported in Materials & Methods and Limitations and Future Directions. Standardized testing procedures, calibrated equipment, certified examiners, sex-specific standardization of fitness components, and consistent scoring procedures were used to reduce measurement and scaling bias. Potential selection bias and residual confounding due to unavailable health-screening and behavioral variables are formally acknowledged in the Limitations section. |
| Study size | 10 | Explain how the study size was arrived at  Reported in Materials & Methods (Participants). The analytic sample consisted of all eligible students with complete de-identified institutional records for sex, academic grade, age, height, body mass, BMI, and all six required fitness components. |
| Quantitative variables | 11 | Explain how quantitative variables were handled in the analyses. If applicable, describe which groupings were chosen and why  Reported in Materials & Methods (Anthropometric Measures; Physical Fitness Index; Statistical Analysis; Nonlinear Modeling). BMI was analyzed both categorically using Chinese adult BMI criteria and continuously in quadratic models. Fitness components were standardized within sex to construct PFI. Sprint and endurance-run times were reverse-scored before standardization. PFI was analyzed as a continuous outcome. |
| Statistical methods | 12 | (*a*) Describe all statistical methods, including those used to control for confounding  Reported in Materials & Methods (Statistical Analysis; Nonlinear Modeling). The manuscript describes descriptive statistics, two-way ANOVA, Bonferroni-adjusted post-hoc comparisons, assumption testing, effect-size reporting using partial eta squared, Pearson correlation analysis, VIF, KMO, Bartlett’s test, PCA, and sex- and grade-specific quadratic regression models. No confounder-adjusted models were conducted because relevant behavioral and health-related confounders were not available in the dataset; this limitation is acknowledged. |
|  |  | (*b*) Describe any methods used to examine subgroups and interactions  Reported in Materials & Methods (Statistical Analysis; Nonlinear Modeling). Subgroup and interaction analyses included sex × grade ANOVA, sex-stratified BMI category × grade ANOVA, and sex- and grade-specific quadratic models |
|  |  | (*c*) Explain how missing data were addressed  Reported in Materials & Methods (Participants). Only records with complete anthropometric data and complete results for all six required fitness components were included. Records with incomplete or unavailable required variables were not included in the analytic dataset. |
|  |  | (*d*) If applicable, describe analytical methods taking account of sampling strategy  Not applicable, as no complex sampling strategy was used. |
|  |  | (*e*) Describe any sensitivity analyses  Not applicable, as sensitivity analyses were not conducted. |
| Results | | |
| Participants | 13* | (a) Report numbers of individuals at each stage of study—eg numbers potentially eligible, examined for eligibility, confirmed eligible, included in the study, completing follow-up, and analysed  Reported in Materials & Methods (Participants) and Results (Participant Characteristics), stating that 16,292 university students were included in the final analysis, including 8,278 females and 8,014 males. |
|  |  | (b) Give reasons for non-participation at each stage  Partly addressed in Materials & Methods (Participants). The manuscript states that the analysis was restricted to complete valid de-identified records. Detailed numbers and reasons for incomplete or invalid records were not available in the dataset, and this issue is acknowledged as a limitation. |
|  |  | (c) Consider use of a flow diagram  Not applicable. A flow diagram was not used because the study was based on de-identified institutional complete-record data rather than prospective recruitment. |
| Descriptive data | 14* | (a) Give characteristics of study participants (eg demographic, clinical, social) and information on exposures and potential confounders  Reported in Results (Participant Characteristics) and Table 1, which present demographic, anthropometric, BMI, and physical fitness characteristics by sex and academic grade. The manuscript also states that potential behavioral and health-related confounders were not available. |
|  |  | (b) Indicate number of participants with missing data for each variable of interest  The analytic dataset included only complete records for the required variables. Detailed missing-data counts for excluded or invalid records were not available and are acknowledged as a limitation. |
| Outcome data | 15* | Report numbers of outcome events or summary measures  Reported in Results and Tables 1–4, which present summary measures for anthropometric variables, physical fitness components, PFI, BMI categories, ANOVA results, and BMI–PFI associations. |
| Main results | 16 | (*a*) Give unadjusted estimates and, if applicable, confounder-adjusted estimates and their precision (eg, 95% confidence interval). Make clear which confounders were adjusted for and why they were included  Reported in Results. The manuscript presents unadjusted group comparisons, ANOVA results, partial eta squared values, correlation coefficients, and quadratic model findings. Confounder-adjusted estimates were not reported because potential behavioral and health-related confounders were not available. |
|  |  | (*b*) Report category boundaries when continuous variables were categorized  Reported in Materials & Methods (Anthropometric Measures), where BMI categories are defined according to Chinese adult BMI criteria: underweight, normal weight, overweight, and obese. |
|  |  | (*c*) If relevant, consider translating estimates of relative risk into absolute risk for a meaningful time period  Not applicable. Relative risk estimates were not calculated. |
| Other analyses | 17 | Report other analyses done—eg analyses of subgroups and interactions, and sensitivity analyses  Reported in Results. Additional analyses included sex-stratified BMI category × grade ANOVAs, sex- and grade-specific quadratic models, Pearson correlations among fitness components, VIF analysis, KMO and Bartlett’s test, and exploratory PCA. Sensitivity analyses were not conducted. |
| Discussion | | |
| Key results | 18 | Summarise key results with reference to study objectives  Reported in the first paragraph of the Discussion, which summarizes sex- and grade-related differences, BMI-category associations with PFI, the concave BMI–PFI pattern, and the correlation structure of the PFI in relation to the study objectives. |
| Limitations | 19 | Discuss limitations of the study, taking into account sources of potential bias or imprecision. Discuss both direction and magnitude of any potential bias.  Reported in Limitations and Future Directions. The manuscript discusses the cross-sectional design, absence of direct body-composition measures, possible BMI misclassification, potential selection bias due to complete-record inclusion, lack of health-screening and behavioral confounders, absence of external validation of the PFI, limitations of quadratic models, and restricted generalizability. The direction and magnitude of some potential biases could not be estimated because information on incomplete records and unmeasured confounders was unavailable. |
| Interpretation | 20 | Give a cautious overall interpretation of results considering objectives, limitations, multiplicity of analyses, results from similar studies, and other relevant evidence.  Reported in Discussion, Practical Implications, Limitations and Future Directions, and Conclusions. The manuscript interprets the findings as cross-sectional associations rather than causal effects, discusses effect-size magnitude, compares findings with previous literature, and avoids treating model-derived BMI peaks as physiological optima or recommended BMI targets. |
| Generalisability | 21 | Discuss the generalisability of the study results.  Reported in Limitations and Future Directions and Conclusions. The manuscript states that the sample was drawn from a specific university population and regional testing context in China and that findings should be generalized cautiously to students from other universities, regions, countries, age groups, or educational systems. Replication in multi-campus and demographically diverse cohorts is recommended. |
| Other information | | |
| Funding | 22 | Give the source of funding and the role of the funders for the present study and, if applicable, for the original study on which the present article is based.  Reported in the Funding section, which states the funding sources, grant numbers, and that the funders had no role in study design, data collection and analysis, decision to publish, or preparation of the manuscript. |

*Give information separately for exposed and unexposed groups.

**Note:** An Explanation and Elaboration article discusses each checklist item and gives methodological background and published examples of transparent reporting. The STROBE checklist is best used in conjunction with this article (freely available on the Web sites of PLoS Medicine at http://www.plosmedicine.org/, Annals of Internal Medicine at http://www.annals.org/, and Epidemiology at http://www.epidem.com/). Information on the STROBE Initiative is available at www.strobe-statement.org.
